# Virulence and pathogenicity of three *Trypanosoma brucei rhodesiense* stabilates in a Swiss white mouse model

**DOI:** 10.4102/ajlm.v4i1.137

**Published:** 2015-10-05

**Authors:** Christopher Kariuki, John M. Kagira, Victor Mwadime, Maina Ngotho

**Affiliations:** 1 Institute of Primate Research, Kenya; 2 Jomo Kenyatta University of Agriculture and Technology, Kenya

## Abstract

**Background:**

A key objective in basic research on human African trypanosomiasis (HAT) is developing a cheap and reliable experimental model of the disease for use in pathogenesis and drug studies.

**Objective:**

With a view to improving current models, a study was undertaken to characterise the virulence and pathogenicity of three *Trypanosoma brucei rhodesiense* stabilates, labelled as International Livestock Research Institute (ILRI)-2918, ILRI-3953, and Institute of Primate Research (IPR)-001, infected into Swiss white mice.

**Methods:**

Swiss white mice were infected intraperitoneally with trypanosomes and observed for parasitaemia using wet blood smears obtained by tail snipping. Induction of late-stage disease was undertaken using diminazene aceturate (40 mg/kg, Berenil) with curative treatment done using melarsoprol (3.6 mg/kg, Arsobal).

**Results:**

The prepatent period for the stabilates ranged from three to four days with mean peak parasitaemia ranging from Log_10_ 6.40 to 8.36. First peak parasitaemia for all stabilates varied between six and seven days post infection (DPI) followed by secondary latency in ILRI-2918 (15–17 DPI) and IPR-001 (17–19 DPI). Survival times ranged from six DPI (ILRI-3953) to 86 DPI (IPR-001). Hindleg paresis was observed in both ILRI-3953 (at peak parasitaemia) and ILRI-2918 (after relapse parasitaemia). Mice infected with IPR-001 survived until 54 DPI when curative treatment was undertaken.

**Conclusions:**

This study demonstrated that the stabilates ILRI-2918 and ILRI-3953 were unsuitable for modelling late-stage HAT in mice. The stabilate IPR-001 demonstrated the potential to induce chronic trypanosomiasis in Swiss white mice for use in development of a late-stage model of HAT.

## Introduction

Human African trypanosomiasis (HAT), or sleeping sickness, is a parasitic infectious disease caused by infection with the haemoprotozoans *Trypanosoma brucei gambiense* (chronic form/Gambian HAT) or *T. b. rhodesiense* (acute form/Rhodesian HAT), two morphologically-identical but epidemiologically-distinct subspecies of *T. brucei*. Both parasites are transmitted cyclically by haematophagous tsetse flies of the genus *Glossina* and are restricted to discrete foci within sub-Saharan Africa.^1,2,3,4,5,6^ HAT is the archetype of a neglected disease, affecting the poorest people in Africa.^7^ The disease runs an intricate course which can result in death if not promptly managed.^8^ The disease pathology consists of two distinct stages. During the early (haemolymphatic) stage, trypanosomes are present in the lymphatic fluid and blood, as well as the extravascular spaces of most organs. In the late (meningoencephalitic) stage, parasites are detected within the central nervous system (CNS), where they cannot be treated by drugs that are incapable of crossing the blood-brain barrier.^3^

One of the major objectives in current basic research on HAT is the development of a cheap and reliable experimental model of the disease that can be utilised to both increase our understanding of the disease and allow for the development of drug regimens for the management of late-stage HAT.^9^ However, the development of such a model has proven to be difficult as most *T. brucei* subspecies tend to cause an acute rather than chronic infection in experimental animals.^10,11^

Presently, drug efficacy trials against late-stage HAT are performed in both murine models (*Mus musculus*) and in non-human primates such as vervet monkeys (*Chlorocebus aethiops*).^9,12,13,14,15,16,17^ Whilst the vervet monkey model of Rhodesian HAT has been widely used in a number of drug evaluation and pathogenesis studies,^9^ it possesses unique disadvantages for the purposes of basic research. Primates represent an expensive and ethically-challenging model, whose use is only justifiable under the auspices of avoiding unnecessary human involvement in most studies. On the other hand, most mouse models of Rhodesian HAT have been extensively developed using the non-human infective *T. b. brucei*, which may possess dissimilar pathogenicity and drug susceptibility profiles when compared to the human-infective *T. b. rhodesiense* or *T. b. gambiens e*.^11^ In particular, these mouse models, being non-human infective and parasite-based, may not depict accurately the CNS involvement by human-infective parasites.^10,11,12,15^ Thus, mouse models for Rhodesian HAT, particularly those based on *T. b. rhodesiense*, are essential in order to provide a more correct depiction of the disease's pathogenicity and drug susceptibility profiles.^11^ As different parasite strains from a particular subspecies may have dissimilar disease progression, it is of interest to perform preliminary studies that compare a number of strains in order to have a proper baseline for future studies involving the selected parasite strains.

This study aimed to characterise the virulence and pathology of three *T. b. rhodesiense* stabilates, International Livestock Research Institute (ILRI)-2918, ILRI-3953, and Institute of Primate Research (IPR)-001, the target parasites for establishment of the rodent and primate models of HAT, in outbred Swiss white mice. To the best of the authors’ knowledge, these parasites have not yet been evaluated comparatively.

## Methods

### Animals

As the pathogenicity and virulence of two of the trypanosome subspecies used were known (ILRI-2918 and ILRI-3953), available data from previous studies were used to estimate the sample size per group.^11,18,19,20^ Swiss white mice of either sex (*n* = 108), weighing 25–30 grams, were obtained from the rodent facility at the IPR and provided with mouse feed (Mice cubes®, Unga Feeds, Nairobi, Kenya) and water *ad libitum*. The mice were kept in 14 cm x 30 cm x 15 cm Macrolone cages. They were maintained at an ambient temperature of between 20 and 25^°^C and wood shavings (various timber mills, Embulbul, Kajiado, Kenya) were used as bedding.

### Trypanosomes

Stabilate ILRI-2918 was clonally derived from Kenya Trypanosomiasis Research Institute (KETRI)-2772, which was isolated from a natural infection in a human in Alupe, Busia, Kenya and designated 2772.^18^ Stabilate ILRI-3953 was originally referred to as Uganda Trypanosomiasis Organisation (UTRO)-310185, which was isolated from a naturally infected human in Bugiri, Uganda in 1982. The parasite has been passaged in mice and designated ILRI-3953.^19,20^ Stabilate IPR-001 was obtained from a cerebrospinal fluid sample from an infected human in Bugiri, Uganda. After three passages in outbred Swiss white mice at the IPR, the stabilate was designated IPR-001.

For the current study, all stabilates were obtained from storage in liquid nitrogen (–196 °C), thawed and diluted with phosphate saline glucose (PSG) buffer (pH 8.0), then injected intraperitoneally (0.2 ml each) into three Swiss white female mice that were immunosuppressed via irradiation with caesium chloride at 600 rads for five minutes. Upon the onset and rise of parasitaemia, the imunosuppressed mice were euthanised and their blood collected via cardiac puncture. The collected blood was then diluted to a parasitaemia of approximately 10^4^ trypanosomes/0.2 mL using PSG, after which the diluted blood was injected intraperitoneally (ip; 0.2 mL each) into the 108 mice included in the study, as described below. Six mice were used as positive controls and injected with PSG buffer only (0.2 mL ip).

### Experimental design

The experiment was designed and executed as indicated in Table 1. After infection, experimental mice were observed daily for parasitaemia using wet blood smears obtained by tail snipping. At the same time, thin blood smears were also prepared and Giemsa stained for observation of pleomorphism via microscopy, as described by Kagira et al.^11^ The parasitaemia was then estimated using the method described by Herbert and Lumsden.^21^

**TABLE 1:** Experimental design.

Variables	ILRI-2918	ILRI-3953	IPR-001
Number of mice	27	38	43
Treatment	- Subcurative treatment^†^	- Euthanasia and pathology	- Subcurative treatment^†^
	- Curative treatment^‡^		- Curative treatment^‡^
	- Euthanasia and pathology		- Euthanasia and pathology

†, Diminazene aceturate (40 mg/kg ip, Berenil®).

‡, Melarsoprol (3.6 mg/kg ip, Arsobal®).

For both ILRI-2918 and IPR-001-infected mice, all surviving mice (both experimentally infected and controls) were treated with diminazene aceturate (40 mg/kg ip, Berenil®, Intervet, South Africa) at 21 days post infection (DPI) as described previously by Kagira et al.^11^ At this stage, the parasites were assumed to have invaded the CNS. The diminazene aceturate therefore only clears the haemolymphatic trypanosome complement, because it cannot cross the blood-brain barrier in sufficient quantities to clear the CNS. Mice were then observed daily for relapse parasitaemia using wet smears obtained via tail snipping. Relapse detection sensitivity was enhanced by examining the collected blood using the Woo method.^22^ Upon relapse of parasitaemia, all surviving mice (both experimentally infected and controls) were treated with melarsoprol (3.6 mg/kg ip, Arsobal®, Sanofi-Aventis, Paris, France) for four days consecutively. Six mice per stabilate were euthanised at set time points (DPI 7, 14 and 21). All their major organs (heart, liver, spleen, brain, lungs and kidneys) were harvested and perfused with citrate saline buffer via the left ventricle through the hepatic portal vein until all the blood was completely drained. They were then preserved in 10% neutral buffered formalin for histopathology.

### Statistical analyses

Data were managed and analysed using MS Excel (Microsoft Excel® 2007, Microsoft Corporation, Redmond, Washington, United States). The means for parasitaemia and parasite morphology were calculated using the AVERAGE function in MS Excel and variability was calculated using the standard error of the mean function in Microsoft Excel, ‘= (STDEV(A1:A2))/(SQRT(COUNT(A1:A2))’, where A1 represents the first value in the series and A2 represents the last value in the series.

### Ethical considerations

All experiments were carried out at the IPR in Karen, Nairobi, Kenya. The experiments were conducted in accordance with protocols approved and authorised by the Institutional Review Committee of the Institute.

## Results

### Parasitaemia and gross clinical observations

**FIGURE 1:**
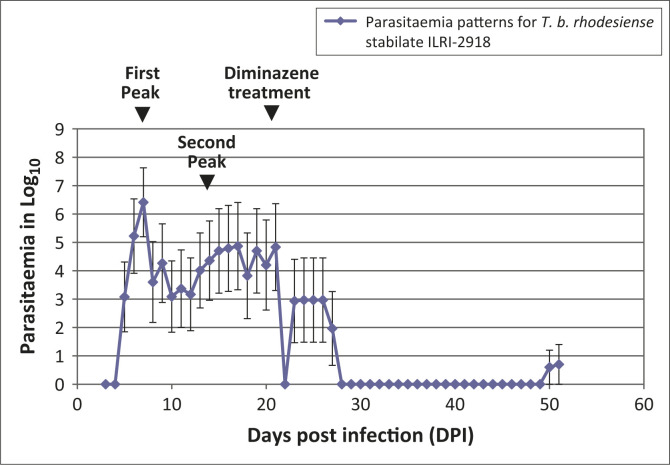
Mean parasitaemia pattern for *T. b. rhodesiense* ILRI-2918 infection in Swiss white mice. The first peak occurred at seven DPI; the second peak occurred at 17 DPI. Mean parasitaemia at the first peak was estimated at Log_10_ 6.4. Mean parasitaemia at the second peak was estimated at Log_10_ 4.87. Following treatment at 21 DPI with diminazene aceturate (40 mg/kg ip, Berenil®, Intervet, South Africa), parasitaemia dropped below microscopically-detectable levels between 22 and 28 DPI. Relapse parasitaemia was observed on 50 DPI. The parasitaemia at the onset of relapse was Log_10_ 0.6.

In ILRI-2918-infected mice, patency was demonstrated at five DPI (Figure 1). This was estimated at Log_10_ 3.07. All of the patent mice were active and had smooth coats at the onset of patency. First peak parasitaemia was observed at seven DPI (Figure 1) and the mean peak parasitaemia was estimated at Log_10_ 6.4. Secondary latency was observed at 17 DPI (Figure 1), with mean peak parasitaemia estimated at Log_10_ 4.87. Following treatment at 21 DPI with diminazene aceturate, parasitaemia dropped below microscopically-detectable levels between 22 and 28 DPI (Figure 1). Relapse parasitaemia was observed on 50 DPI. The parasitaemia at the onset of relapse was Log_10_ 0.6.

From 10 DPI, the mice began to develop ruffled coats, lower appetite and lower activity levels. These symptoms occurred at varying levels of parasitaemia, with some groups experiencing the symptoms during the rising phase of parasitaemia and others during the dropping phase of the first parasitaemia peak. Peri-orbital oedema also occurred during the rising and dropping phases of parasitaemia, with three mice having one or both eyes shut at all times. These symptoms persisted until the second rising phase of parasitaemia, at which time they disappeared and the mice appeared active and once again had smooth coats. At 26 and 27 DPI, hindleg paresis was observed in two mice. These mice were euthanised and their major organs collected for histopathology. At 27 DPI, after treatment with diminazene aceturate at 21 DPI, the surviving mice again had lowered activity and ruffled coats. The prognosis of these mice, however, improved steadily and the clinical symptoms slowly dissipated, disappearing by 31 DPI. At 50 DPI, all surviving mice relapsed into parasitaemia at Log_10_ 0.6 and at 51 DPI, the mice succumbed to hindleg paresis and were subsequently euthanised.

**FIGURE 2:**
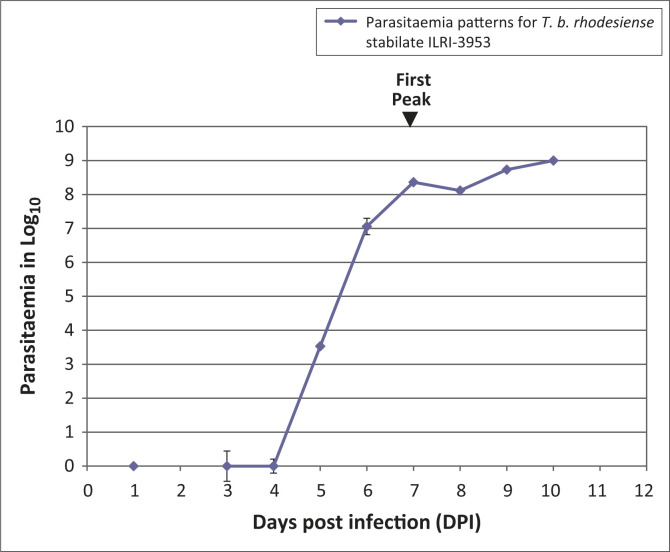
Mean parasitaemia pattern for *T. b. rhodesiense* ILRI-3953 infection in Swiss white mice. The first peak occurred at seven DPI. Mean parasitaemia at the first peak was estimated at Log_10_ 8.36. All mice were found dead in their cages or were euthanised by 10 DPI.

In ILRI-3953-infected mice, patency was demonstrated from three DPI, with peak parasitaemia observed at seven DPI (Figure 2). This was estimated at Log_10_ 8.36. All of the patent mice were active and had smooth coats at the onset of patency. Mice began to develop ruffled coats, lower activity level and paresis from seven DPI. One mouse also exhibited peri-orbital oedema at peak parasitaemia (Log_10_ 8.4; seven DPI). As the parasitaemia rose, mice developed hindleg paresis and were found dead in their cages, with some deaths occurring at six DPI. By 10 DPI, all experimental mice had been euthanised or were found dead in their cages, necessitating a termination of the experiment on this isolate.

**FIGURE 3:**
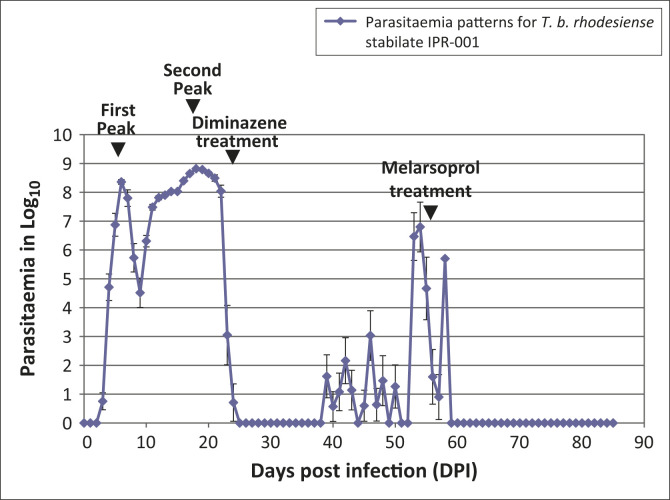
Mean parasitaemia pattern for *T. b. rhodesiense* IPR-001 infection in Swiss white mice. The first peak occurred at six DPI; the second peak occurred at 18 DPI. Mean parasitaemia at the first peak was estimated at Log_10_ 8.36. Mean parasitaemia at the second peak was estimated at Log_10_ 8.81. Following treatment at 21 DPI with diminazene aceturate (40 mg/kg i.p., Berenil®, Intervet, South Africa), parasitaemia dropped below microscopically-detectable levels between 22 and 25 DPI. Relapse parasitaemia was observed to occur at 39 DPI (Log_10_ 1.62) and peaked at 54 DPI (Log_10_ 6.8). Following treatment with melarsoprol (3.6 mg/kg ip, Arsobal®, Sanofi-Aventis, Paris, France), parasitaemia was again observed to drop below microscopically-detectable levels until the termination of the experiment at 86 DPI.

In IPR-001-infected mice, patency was demonstrated from three to five DPI (Figure 3). All of the patent mice were active at the onset of patency. The first peak of parasitaemia was observed at six DPI (Log_10_ 8.36) and the second peak parasitaemia occurred at 18 DPI (Log_10_ 8.81). Ruffled coats, lower appetite and lower activity level were observed from as early as the first parasitaemia peak. After treatment with diminazene aceturate, parasitaemia dropped below microscopically-detectable levels and clinical symptoms abated. Relapse parasitaemia occurred at 39 DPI (Log_10_ 1.62) and peaked at 54 DPI (Log_10_ 6.8). Following treatment with melarsoprol, parasitaemia was again observed to drop below microscopically-detectable levels until the termination of the experiment at 86 DPI.

### Parasite virulence and mouse survival

**FIGURE 4:**
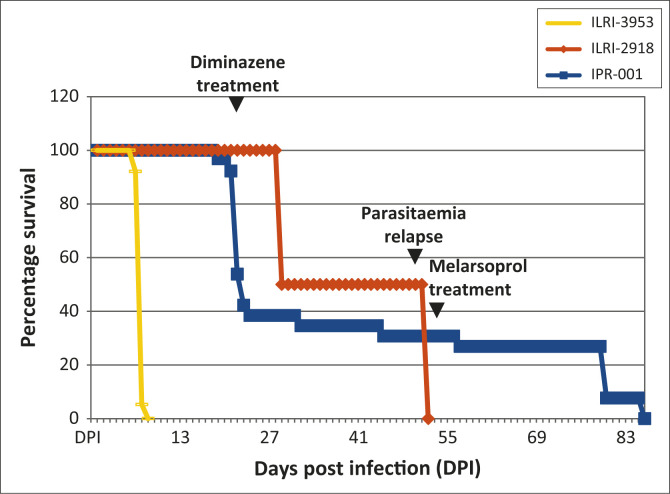
Mean survival for ILRI-2918, ILRI-3953 and IPR-001 infected mice. In ILRI-2918 infected mice, survival was 100% until after treatment with diminazene aceturate at 21 DPI, whereupon 50% developed hindleg paresis and were euthanised with the rest of the population succumbing to the parasite after parasitaemia relapse. In ILRI-3953 infected mice, mortality was 100% by 10 DPI. In IPR-001 infected mice, survival was 97% before treatment with diminazene aceturate at 21 DPI, dropping and stabilising at 38% (24–31 DPI), followed by 35% (32–44 DPI), then 31% (45–56 DPI) and finally stabilising after treatment with melarsoprol to 27% (57–79 DPI). All mice surviving after the treatment were euthanised.

In ILRI-2918-infected mice, survival was constant (100%) up to and after treatment with diminazene aceturate at 21 DPI (Figure 4). Upon treatment, mice developed hindleg paresis (50% of the experimental population at the time), at which time they were euthanised. Upon relapse of parasitaemia, the remaining mice succumbed to the parasite, thereby causing termination of the experiment.

In ILRI-3953-infected mice, mortality increased with the rise in parasitaemia, with mice rendered moribund as the experiment progressed. By 10 DPI, all mice were dead in their cages or had been euthanised after hindleg paresis was observed.

In IPR-001-infected mice, mortality occurred even before treatment with diminazene aceturate at 21 DPI. Approximately 3% of the experimental population succumbed to the stabilate's parasitaemia, with 97% surviving before treatment with diminazene aceturate at 21 DPI. The survival dropped further through 38% (24–31 DPI), to 35% (32–44 DPI), then 31% (45–56 DPI), before finally stabilising after treatment with melarsoprol to 27% (57–79 DPI), whereupon it remained stable until the end of the experiment. At the end of the experiment, the surviving mice were euthanised and their organs harvested for histopathology.

### Parasite morphology

Examination of the Giemsa-stained thin blood smears indicated that pleomorphism occurred in two of the parasite strains used, namely, ILRI-2918 and IPR-001. In these stabilates, two trypomastigote forms – long slender and short stumpy – were observed during the course of infection. From five DPI in ILRI-2918 and three DPI in IPR-001, there was a predominance of long slender trypomastigotes (97.4% in ILRI-2918 and 83.7% in IPR-001), with few short stumpy trypomastigotes observed (2.6% in ILRI-2918 and 16.3% in IPR-001). For both strains, observation of the different morphological forms ceased at 23 DPI after treatment with diminazene aceturate at 21 DPI.

**FIGURE 5:**
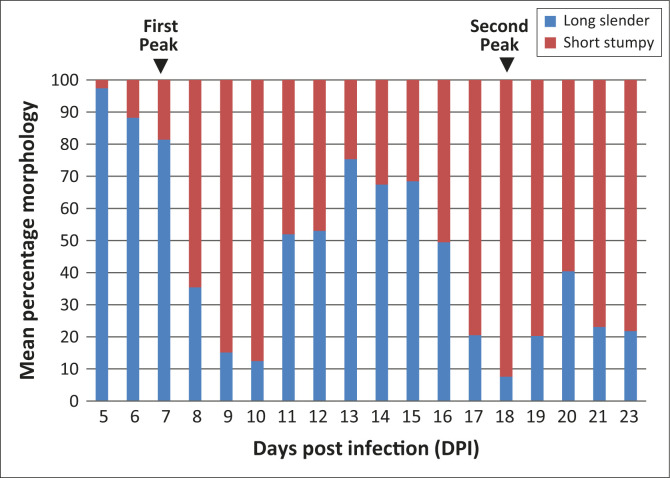
Parasite morphology pattern for ILRI-2918 infection in Swiss white mice. During the first parasitaemia peak at seven DPI, there was an increase in short stumpy forms (18.6%) with a decline in long slender forms (83.7%). At the second peak at 17–19 DPI, there was a predominance of short stumpy forms (up to 92.4%) and a decline of long slender forms (7.6%).

In ILRI-2918-infected mice, there was an increase in short stumpy forms (18.6%) with a decline in long slender forms (83.7%) (Figure 5) during the first parasitaemia peak at seven DPI, compared to the populations at the beginning of the observation period. At eight DPI, there was a great increase in the number of short stumpy trypomastigotes (64.6%), with a decline in the long slender forms (35.4%). At the second peak (at 17–19 DPI), there was a predominance of short stumpy forms (up to 92.4%) and a decline in long slender forms (7.6%).

**FIGURE 6:**
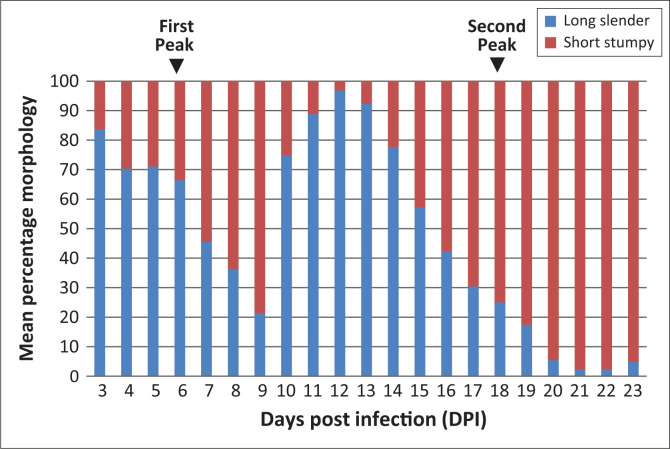
Parasite morphology pattern for IPR-001 infection in Swiss white mice. During the first peak at six DPI, there was a notable increase in the number of short stumpy trypomastigotes (33.6%) and a decline in the long slender forms (66.4%) compared to the populations at the beginning of the observation period. At the second peak at 18 DPI, there was a predominance of short stumpy forms (74.9%) and a decline of long slender forms (25.1%).

In IPR-001-infected mice, during the first peak at six DPI, there was a notable increase in the number of short stumpy trypomastigotes (33.6%) and a decline in the long slender forms (66.4%) compared to the populations at the beginning of the observation period (Figure 6). In this isolate as well, at seven DPI, there was a great increase in the number of short stumpy trypomastigotes (54.4%) and a decline in the long slender forms (45.6%). At the second peak, at 18 DPI, there was a predominance of short stumpy forms (74.9%) and a decline of long slender forms (25.1%). However, the greatest increase in short stumpy forms during the IPR-001 infection occurred between 20 and 23 DPI (from 94.6% – 95.2%).

### Histopathological findings

**FIGURE 7:**
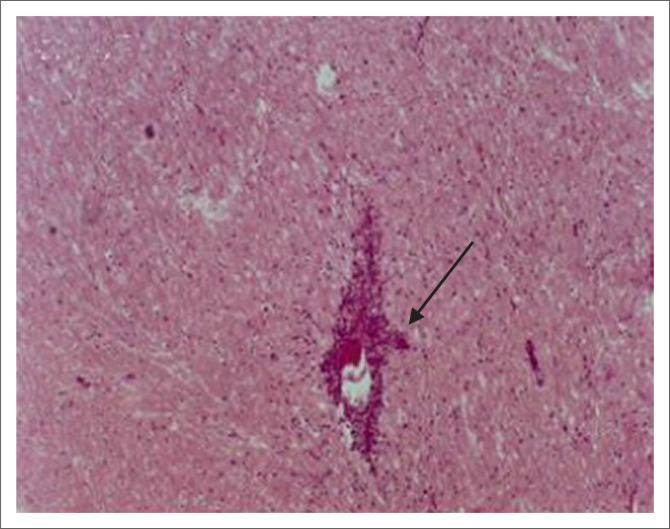
Perivascular cuffing (indicated by the shaded arrow) in a parasite-relapse mouse brain sample.

Gross anatomy observation revealed enlargement of the spleen and hyperaemia in the major organs. Histopathological observations of the brain included discrete inflammation, with the foci of meningitis characterised by progressive mononuclear cell infiltration, perivascular cuffing (Figure 7) and hyperaemia of meningeal blood vessels. These observations were seen to progress over time in all observed samples from experimental mice. Infiltration of the brain parenchyma with mononuclear cells was rare and only began to be prominent in the late stages of infection. The choroid plexus was prominent in infected mice and contained inflammatory exudates, mainly of mononuclear cells.

In all groups of experimental mice, most organs were characterised by interstitial mononuclear cell infiltration into the parenchyma and perivascular cuffing. The most affected organs were the liver, spleen, heart and lungs. There were no marked differences between ILRI-2918 and IPR-001-infected mice. The germinal centres of the spleen were active and contained many immature lymphocytes and plasma cells, especially in samples obtained at 14 and 21 DPI, although these cells were reduced in number by 28 DPI. The lung alveoli, as well as the interseptal spaces, were characterised by infiltration with inflammatory exudates and cells, including lymphocytes, macrophages and a few neutrophils. This was more prominent in samples from mice euthanised after 21 DPI. The liver indicated extensive lymphocytic infiltration coupled with necrosis of hepatocytes, especially in mice euthanised after 28 DPI.

## Discussion

Of the three stabilates investigated, we found that two possessed the necessary parasitaemic characteristics of a typical HAT infection. Both ILRI-2918 and IPR-001 had a mean pre-patent period and parasitaemia pattern similar to that reported by other researchers.^11^ The survival times for these two stabilates and the pathological changes they induced in experimental animals demonstrated that these parasite strains are capable of causing a chronic infection. This is a feature particularly useful in models for drug efficacy testing. Some of the symptoms observed in mice infected with ILRI-2918 and IPR-001, particularly peri-orbital oedema, have also been described in higher primate models of HAT.^13,23^ The deaths occurring at 31 DPI for ILRI-2918 and at 21 DPI for IPR-001 could have resulted from an immunological crisis arising after massive trypanolysis occasioned by treatment with diminazene aceturate. The mortality and/or morbidity observed in trypanosome-infected mice could be attributed primarily to self-inflicted damage by a disproportionate immune and/or innate response.^24^ Mortality and morbidity could also be caused by the immuno-depressant capabilities of a trypanosome infection on a mouse strain. This may have led to opportunistic infections, leading to the death of mice infected with a trypanosome strain.^25^

In this study, the parasitaemia pattern showed a predominance of two different morphological types of trypomastigotes, namely, short stumpy and long slender forms. In both ILRI-2918 and IPR-001 infections, there was a predominance of short stumpy forms during or shortly after peak parasitaemia. This phenomenon was observed at both parasitaemia peaks and appeared to be alleviated by treatment with diminazene aceturate treatment. However, for both strains, the situation was reversed during the rising phase of the parasitaemia. In this case, the short stumpy form declined and the long slender form predominated. This observation is in line with what has been reported by others.^11^ Pleomorphism is considered a pre-adaptation to transmission to the tsetse fly, with long slender, multiplicative forms being transformed into short stumpy, non-multiplicative forms capable of survival in the tsetse fly midgut.^26^ This process, which is a host antibody-independent response, is probably mediated by a density-sensing mechanism.^27^

The pathological changes described in most organs appeared to be characteristic of *T. brucei* infections. Unlike most other Trypanozoon genus members, *T. brucei* is particularly tissue invasive, causing cellular infiltration into the parenchyma of most organs.^11,13^ The cellular reactions we observed in most organs showed that a lymphoid immune reaction is critical in the pathogenesis of *T. b. rhodesiense* infections, as described previously by others.^11,13,28^ In the brain, it has been shown that the inflammatory reaction begins as meningitis and progresses into the Virchow-Robin spaces before spreading to the parenchyma.^11^ Whilst the successful induction of the meningoencaphalitic stage was assumed here (because of the relapsing parasitaemia after treatment with diminazene aceturate, which clears only the haemolymphatic component of the parasite), further proof in terms of demonstration of trypanosomes within the CNS, coupled with appearance of anti-trypanosomal antibodies as described by previous studies,^29,30^ would greatly improve the value of the reported model.

In addition, the role played by the particular mouse strain can influence the disease progression and pathology encountered. This has been shown by others, with certain strains such as BALB/c mice showing the greatest susceptibility, whereas C57Bl/6 mice show the greatest tolerance.^25,31^ Given that the mice used in this study were classified as outbred Swiss white, there could be a significant deviation, particularly in the disease pathology, when the model is established with inbred strains.

Most murine models of trypanosomiasis for drug efficacy trials rely on the assumption that trypanosomes have invaded the brain parenchyma by 21 DPI.^10,21^ In this study, the same assumption was made with subcurative treatment with diminazene aceturate. Relapses occurred in both ILRI-2918- and IPR-001-infected mice, coupled with severe meningoencephalitis as has been reported previously.^10,11,32^

### Limitations of the study

As described in the preceding section, there were a number of limitations observed which can be summarised as follows. Firstly, the strain of mouse greatly influences the disease progression and pathology encountered. There could thus be deviations if the model is established using a different mouse strain, particularly an inbred one. Secondly, the study assumed the occurrence of CNS involvement by relapse of parasitaemia after haemolymphatic clearance with diminazene aceturate, as has been done in previous studies. However, assessment of plasma concentrations of acute-phase protein, C-reactive protein and haptoglobin could also be used as a marker for experimental infections.^16,33^ Thirdly, it is difficult to interpret brain pathology in terms of relapse following treatment with trypanocidals, as they, too, have been shown to induce brain pathology. Another point of contention when using trypanocidals to stage the disease is the danger that subcurative treatment of a parasitic infection may lead to emergence of drug resistance, a position which would complicate the interpretation of efficacy data if the drug under investigation has a similar mode of action.^11^ Finally, the parasites used in this study have not yet been characterised genetically.

### Recommendations

In order to circumvent the preceding limitations, an initial baseline study is required for any novel mouse model utilising the described parasite isolates, incorporating more extensive measures of parasitaemia as well as parasite staging. Full genetic characterisation of the parasites used here is needed in order to ascertain the identity of the isolates during future experiments and in order to account for variations observed in subsequent studies. Because of the heterogenous nature of outbred Swiss mice, there may be significant deviations in results from mouse studies using the same isolates in the future. For this reason, studies using the more homogenous inbred mouse strains, which may not be susceptible to the isolates used here, are recommended in order to establish the late-stage HAT model.

## Conclusion

In conclusion, this study investigated infection with three different *T. b. rhodesiense* strains, ILRI-2918, ILRI-3953 and IPR-001, which led to the occurrence of acute, hyper-acute and chronic infections, respectively. ILRI-2918 showed indications of causing a chronic infection in mice; however, its relapse parasitaemia induced hindleg paresis and death. This strain of the parasite was therefore classified as causing an acute infection in mice and was regarded by the investigators as being unsuitable for the development of a late-stage model of HAT.

ILRI-3953 was observed to be highly virulent, with all the experimental mice found dead in their cages or humanely euthanised by 10 DPI. Upon further passaging in immunocompetent Swiss white mice (unpublished data), the parasite showed no indications of reducing its virulence. Therefore, the parasite was classified as hyper-acute and unsuitable for the development of a late-stage model of HAT.

IPR-001, a field-isolated parasite, showed indications of causing a chronic infection in mice. The first peak of parasitaemia rose to Log_10_ 8.36 at six DPI. After subcurative treatment, mice relapsed and survived in good physical condition/health until curative treatment. This parasite was therefore classified as causing chronic infection and is proposed as the parasite of choice for further work in developing a late-stage model of HAT.

## Trustworthiness

In as far as the authors are concerned, and in view of the outcome of the *T. b. rhodesiense* infections in the mouse studies, the results obtained in this study appear to compare well with those arising from *T. b. brucei* and other related studies.

### Reliability and validity of the research

The authors consider the experimental design of this study to be reliable and valid for the purpose of establishing a mouse model in outbred Swiss white mice. The procedures utilised in this study have been tested and validated in other studies as cited in this article.
